# Novel High-Throughput Screen Identified S100A4 Inhibitors for Anti-Metastatic Therapy

**DOI:** 10.7150/ijbs.113805

**Published:** 2025-07-11

**Authors:** Paul Curtis Schöpe, Nina Heisterkamp, Devin Schütz, Guido Mastrobuoni, Kerstin Putzker, Ulrike Uhrig, Wolfgang Walther, Stefan Kempa, Marc Nazaré, Dennis Kobelt, Ulrike Stein

**Affiliations:** 1 Experimental and Clinical Research Center, Charité - Universitätsmedizin Berlin, and Max-Delbrück-Center for Molecular Medicine, Robert-Rössle-Str. 10, 13125 Berlin, Germany.; 2 The European Molecular Biology Laboratory, EMBL, Meyerhofstraße 1, 69120, Heidelberg, Germany.; 3 German Cancer Consortium (DKTK Partnersite Berlin), Deutsches Krebsforschungszentrum (DKFZ), Im Neuenheimer Feld 280, 69120 Heidelberg, Germany.; 4 Proteomic and Metabolomics Platform, Berlin Institute for Medical Systems Biology (BIMSB), Max Delbrück Center for Molecular Medicine in the Helmholtz Association, Berlin, Germany.; 5 Leibniz-Forschungsinstitut für Molekulare Pharmakologie, FMP, Robert-Rössle-Str. 10, 13125 Berlin, Germany.

**Keywords:** colorectal cancer, metastasis, S100A4, HTS, targeted therapy

## Abstract

Colorectal cancer (CRC) metastasis continues to account for a substantial proportion of cancer-related deaths worldwide. Calcium-binding protein S100A4 is a known executor of CRC metastasis. S100A4 has been correlated to metastasis formation in the past, and therefore pharmaceutical intervention reduces the metastatic phenotype. Herein, a high-throughput screen (HTS) of 105,600 compounds from the EMBL screening library using an S100A4 promoter-driven luciferase construct transfected into HCT116 cells identified novel compounds for S100A4 transcriptional inhibition. The most promising inhibitors identified were tested for S100A4 transcriptional inhibition, their impact on wound healing, migration, proliferation and viability of cancer cells. Subsequently, the leading candidate E12 was tested *in vivo* in a xenograft mouse model (HCT116/CMVp- Luc). After several testing rounds, E12 a 2-(4-fluorobenzenesulfonamido)benzamide-based compound showed the strongest inhibition of S100A4 expression at mRNA (EC_50_ < 1 µM; 48 h) and protein level and concomitant restriction of metastatic abilities in two CRC cell lines with a tolerable viability reduction. *In vivo*, a reduction in metastasis formation was demonstrated, displayed by reduced overall bioluminescence of tumors and human satellite DNA in the liver of treated mice. This study exhibited E12's promising potential for S100A4 targeted metastasis inhibition therapy to improve the outcome of metastasized CRC patients.

## 1. Introduction

Colorectal cancer (CRC) is evidently an unmet clinical need - this public health emergency is reflected by the fact that CRC is the third most common cancer globally, and further the second-leading cause of cancer related deaths worldwide 1. It is well-defined that metastasis is the most lethal attribute of CRC. The truth lies in the statistics: the cancer cell dissemination process clearly reduces patient survival by accounting for 80.2% of all CRC deaths 2. Furthermore, those diagnosed in not distantly metastatic stages I-III have a 5-year survival rate (YSR) of 82.4%, whereas metastatic stage IV patients exhibit a mere 15.7% 3. Notably, not even patients in stages I-III avoid this deadly spread: approximately 20% of patients initially diagnosed in stages I-III will experience distant metachronous metastasis normally within the first 3 years of their diagnoses 4. This shadow of metastasis that haunts CRC diagnoses emphasizes the dire need to refine current treatment methods to target metastasis-associated molecular processes and thereby prolong patient's lives.

The S100 calcium-binding protein family member S100A4 represents a potent executer of CRC metastasis 5-9, and thus represents an excellent target for therapeutic interference. S100A4 possesses a low molecular weight of 11.7 kDa 10 and is highly expressed in tumors of various entities including colorectal, breast, pancreatic, lung, esophageal, medulloblastoma and gastric cancer 11-17. It is moreover considered an invaluable clinical prognostic biomarker, with high S100A4 expression levels being associated with reduced patient survival 15-17. Furthermore, this metastasis-executing gene contains a Transcription Factor 4 (TCF4) binding site in its promoter region that results in it being a target of the canonical Wnt-signaling pathway through its binding with the heterodimeric β-catenin/TCF complex 9. This signaling pathway is aberrant in > 90% of CRC cases, making S100A4 a pertinent target when treating CRC 18. S100A4 fuels metastasis through a variety of mechanisms: firstly, S100A4 can bind extracellularly to RAGE 19 and TLR4 receptors 20, thus driving cell survival, hypoxia mitigation and chronic inflammation. Furthermore, S100A4 activity intracellularly includes binding to non-muscle myosin 2A, tropomyosin and F-actin to drive cellular motility 21-25, increasing matrix metalloproteinase production to degrade the ECM 26,27 and reducing cellular adhesion molecules such as E-cadherin 28,29. These processes result in enhanced cellular motility through enabled epithelial-to-mesenchymal transition, thus heightening migration and metastasis capabilities. Therefore, restricting S100A4-mediated functions is a promising route to reduce metastasis formation in CRC patients.

In this study, a high-throughput screen (HTS) of 105,600 compounds was conducted by the Chemical Biology Core Facility (CBCF) of the European Molecular Biology Laboratory (EMBL) to identify effective S100A4 transcriptional inhibitors. The HTS employed a human S100A4 promoter-based reporter assay with the construct HCT116/S100A4p-Luc, and thereby identified 15 compounds as the best-performing inhibitors. These were subsequently tested in more detail for their specific S100A4 expression inhibition, metastatic phenotype reduction capacities as well as their impact on cellular viability *in vitro*. Following multiple rounds of testing, the most promising lead E12 was identified as the most effective inhibitor of S100A4 transcription from a series of 2-(4-fluorobenzenesulfonamido)benzamide-based compounds. This compound E12 was then further evaluated *in vivo* in a metastatic CRC model for its ability to reduce the primary tumor, and even more importantly, the extent of the metastatic spread to the liver as primary metastatic site. The ultimate aim of this study was to identify a novel efficient anti-metastatic compound that targets the S100A4 driven metastasis of CRC.

## 2. Material and Methods

### Cell lines and cultivation

Human CRC cell lines HCT116 and SW620, initially purchased from American Type Culture Collection were cultivated in Dulbecco's Modified Eagle's Medium (DMEM; Thermo Fisher Scientific Inc, Waltham) supplemented with 10% fetal bovine serum (vol/vol) (FBS; Capricorn Scientific, Ebersdorfergrund) in a humidified incubator at 37°C with 5% CO_2_. The cells were regularly tested for mycoplasma contamination with MycoAlert mycoplasma detection kit (Lonza, Basel). For identification of transcriptional inhibitors of S100A4, HCT116/S100A4p-Luc cells were generated, expressing the human S100A4 promoter-driven [-1487 to +33 bp upstream of the S100A4 transcriptional start site; kindly provided by Dr. David Allard (Peninsula Medical School, University of Exeter and University of Plymouth, Exeter, UK)] luciferase reporter gene 38. Luciferase expression was regularly controlled by the Steady-Glo Luciferase Assay System (Promega, Madison).

### High-throughput screening (HTS)

One of the largest academic libraries with diverse small and drug like compounds in Germany, maintained at EMBL (CBCF), Heidelberg, Germany was used for a screening campaign. For this screen, compounds, dissolved in dimethyl sulfoxide (DMSO, Roche, Basel) were diluted with water and prepared in 384 well white plates (CulturePlates, Revvity, Waltham) and stored at -20°C until the experiment started. HCT116/S100A4p-Luc were seeded into these 384-well plates (2 × 10^3^ cells/well; 50 µL DMEM without phenol red). This resulted into a final compound concentration of 10 µM. After 72 h of incubation, the cells were lysed, and the luciferase expression was determined by Britelite Plus Reporter Gene Assay System (Revvity, Waltham). The luciferase signal was normalized to the solvent control per plate for each compound. The most active (strongest S100A4 promoter inhibition) representative compounds of 15 hit families were selected for an investigation of the EC_50_ (luciferase signal) and IC_50_ (cell viability, ATPLite 1step kit, Revvity, Waltham) analysis under the same experimental conditions for 24 h, 48 h and 72 h treatments. The selected compounds were freshly bought from Enamine (Table S1). The effect of these compounds was investigated in two independent batches, each performed in triplicates. The HTS was conducted by Kerstin Putzker and Ulrike Uhrig at the CBCF of the EMBL, Heidelberg.

### Compounds and treatments

The compounds identified in the HTS and used for further experiments were received from Enamine (Table S1, Riga). All compounds were purchased as powder and then solubilized in DMSO (Carl Roth, Karlsruhe) to a 10 mM stock solution. Storage took place at -20°C. Each experiment was performed with DMSO as the negative control. The DMSO concentration in all wells was adjusted to the highest compound concentration which bears the highest DMSO concentration.

### Quantitative reverse transcription - polymerase chain reaction (qRT-PCR)

For mRNA-expression analysis, cells were seeded into 24-well plates (1 × 10^5^ cells/well; 500 μL DMEM). Following a 24 h incubation period, the cell medium was aspirated, and 500 µL of compound solution was added to each well. Following the varying treatment durations, total RNA was extracted using the Universal RNA Kit (Roboklon, Berlin) and quantified using a Nanodrop (Peqlab, Erlangen). The reverse transcription of 25 ng RNA into cDNA was done using the cDNA Synthesis Kit (Biozym, Hessisch Oldendorf), according to the manufacturer's protocols in volumes of 10 µl. All cDNA was stored at 4°C for immediate or at -20°C for later use. cDNA was diluted 1:3 with water, qRT-PCR was performed using 2 µl of cDNA with the Blue S'Green qPCR Mix (Biozym, Hessisch Oldendorf). Amplification reaction occurred in a PCR 96-well TW-MT-plate using the LightCycler 480 II (Roche, Basel), according to the manufacturer's recommendations in a volume of 10 µl. Data analyses were performed with the software of LightCycler 480 SW 1.5.1.62 (Roche, Basel). All samples were run in technical triplicates and the corresponding mean values were calculated. Each mean value of the cDNA was normalized to the respective mean value of the DMSO control.

### Western blotting

For protein expression analysis, cells were seeded into 6-well plates (5 × 10^5^ cells/well; 2 mL DMEM/well). Following a 24 h incubation period, the cell media was aspirated, and 2 mL of the prepared compound solution was added to each well. After the respective treatment durations, the cells were washed with PBS, detached via scraping and pelleted at 1000 × g for 5 min. Lysis of the cell pellet was followed by dissolving them in 50 µl of RIPA buffer (composed of 50 mM Tris-HCl [pH 7.5], 150 mM NaCl, 1% Nonidet P-40), supplemented with complete protease inhibitor tablets (Roche, Basel) and incubating them on ice for 30 min, whilst vortexing the samples every 10 min. Lysates were centrifuged at 14.800 × g for 30 min at 4°C, after which the protein-containing supernatant was stored at -20°C. Protein concentrations were determined via the Pierce TM BCA Protein Assay Kit (Thermo Fisher Scientific Inc., Waltham) in order to prepare SDS loading samples containing 10 ng of protein, 100 mM DTT, 1 × NuPage™ LDS sample Buffer (Thermo Fisher Scientific Inc, Waltham), and PBS to a total volume of 10 μL. All samples were heated for 10 min at 95°C before being loaded into the SDS gels (acrylamide content: separation gel 15%, stacking gel 5%), stacked at 70 V for 30 min and separated at 120 V for 90 min. A broad range protein ladder (Spectra Multicolor, Thermo Fisher Scientific Inc., Waltham) was used to indicate the molecular weight of the proteins. The transfer was conducted with a methanol-activated PVDF membrane (Bio-Rad, Hercules) via semi-dry electro blotting employing Bio-Rad's blotting system (25 V and 1.3 A were applied for a duration of 7 min). PVDF membranes were blocked for 1 h with a TBS-T solution with 5% BSA. Membranes were cut into sections containing the S100A4 and ß-actin bands and incubated separately with their respective antibody solutions (rabbit anti-human S100A4 antibody (1:3000; Dako, Glostrup), mouse anti-human ß-actin antibody (1:50.000; Sigma-Aldrich, St. Louis)); diluted in TBS-T with 5% BSA) overnight on a shaker at 4°C. The next day, membranes were washed 8 times for 5 min with TBS-T and then incubated with HRP-conjugated secondary antibodies (for the S100A4 section: goat anti-rabbit IgG antibody (1:10.000; Promega, Madison), for the ß-actin section: goat anti-mouse IgG antibody (1:20.000; Invitrogen, Waltham)) for 1 h at room temperature. Following 12 washing steps, each for 5 min with TBS-T, the Western Bright ECL substrate (Biozym, Hessisch Oldendorf) was added to the membranes. Subsequently Xray films (Kisker Biotech, Steinfurt) were exposed to the membranes. All Western blots were repeated independently three times for each compound.

### MTT assay

To evaluate the effect of the compounds on the cell viability, a 3-(4,5-dimethylthiazol-2-yl)-2,5-diphenyltetrazolium bromide (MTT, Thermo Fisher Scientific Inc., Waltham) assay was conducted, investigating cellular metabolic activity. Therefore, cells were seeded in 96-well plates (1 × 10^4^ cells/well; 100 μL DMEM). Following a 24 h incubation period, the cells were treated by adding 100 µL of initially double-concentrated compound solutions in DMEM. After the respective treatment durations, 20 µL of MTT (Carl Roth, Karlsruhe), dissolved in PBS and sterile filtrated, was added to each well in a final concentration of 0.5 mg/ml. Following a 1 h incubation period at 37°C, the entire media was carefully aspirated, and formazan crystals were solubilized via incubation with 100 µl DMSO for 10 min at room temperature. Quantification of metabolized MTT was executed through colorimetric measurement of absorbance at 560 nm, employing the TECAN^TM^ Fluor Spectra Plus Plate Reader and the internal TECAN^TM^ software (Magellan V 7.0, Tecan, Männedorf). All measured values were normalized to the sample with the lowest compound concentration. All MTT assays were repeated independently at least three times for each compound.

### Wound healing assay

To investigate the migratory capacity of CRC cells *in vitro*, a wound healing assay was performed by first seeding a monolayer of cells (1 × 10^5^ cells/well; 100 μL DMEM) in IncuCyte ImageLock 96-well plate (Essen Bioscience, Ann Arbor). Following a 6 h incubation, the cells were treated by adding 100 µL of initially double-concentrated compound solution. Subsequently, a wound width of 700-800 μM was created in the cellular monolayer via the IncuCyte 96-well WoundMaker Tool (Essen Bioscience, Ann Arbor). Wound closure was observed every 2 h for 72 h using the IncuCyte Live cell imaging system (Essen Bioscience, Ann Arbor), capturing microscopic bright-field images of each well. The IncuCyte Zoom software enabled automated quantification of wound confluence in percentage. The wound healing assay was repeated independently three times for each compound.

### Proliferation assay

For the investigation of cell proliferation under compound treatment, cells were seeded in 96-well plates (1 × 10^4^ cells/well; 100 µL DMEM/well). Following a 24 h incubation period, the cells were treated by adding 100 µL of initially double-concentrated compound solutions. Proliferation was monitored over 72 h using the IncuCyte Live cell imaging system (Essen Bioscience, Ann Arbor), which determined the confluence area by analyzing captured microscopic bright-field images of each well. The IncuCyte Zoom software enabled automated quantification of confluence area in percentage. The proliferation assay was repeated independently three times.

### Boyden-chamber transwell migration assay with flow cytometry

On day 0, 5 × 10^5^ cells were seeded in a 10 mL petri dish with DMEM (10% FBS). Following 24 h seeding time, the cells were starved with 0% FBS DMEM on day 1 and incubated overnight. On day 2, 235 µL of 10% FBS DMEM media containing the relevant compound concentration was added to the bottom chamber of the migration plate, followed by addition of 100 µL of a 0.5% FBS DMEM compound/cell master mix to the upper chamber. The plate was incubated at 37°C for 18 h. On day 3, the bottom chamber media was transferred to a 96-well U-plate for flow cytometry analysis. 100 µL of trypsin was added to the bottom chamber for total removal of any adherent migrated cells. The resulting cell-containing media was transferred to an additional 96-well plate (U-bottom) also for flow cytometry analysis. 96-well U-plates were spun down at 300 × g for 7 min. The supernatant was removed, with the remaining U-well liquid being added to the residual pellet and the spin-down step being repeated. Following the second centrifugation step, the cells were washed in 200 µL fluorescence-activated cell sorting buffer and spun down again at the same conditions. The cells were stained with 1:1000 dilution of 4',6-diamidino-2-phenylindole (DAPI)-violet stain (Thermo Fisher Scientific Inc., Waltham) to FACS buffer respectively. 40 µL of the DAPI-violet dilution was added to each well. Using a multi-pipette, the samples were removed from the wells in doublets and transferred to polystyrene flow cytometry tubes using mechanical force. The BD Biosciences LSRFortessaTM machine (BD Biosciences, Franklin Lakes) was primed 3 times prior to usage. The machine was calibrated using one tube from every concentration. The computational software FlowJo v10.10 (FlowJo LLC, Ashland) was used to analyze the data. The migration assay was repeated independently three times.

### Detection of human satellite DNA in mouse liver

Slices from frozen mouse liver tissues were generated with 10 µm thickness and placed into precooled tubes at -20°C. DNA was isolated after adding lysis buffer and subsequent sonication at 50% for 5 pulses using the DNA/RNA/protein extraction kit (Roboklon, Berlin). The isolated DNA was used for qPCR experiments, which were conducted as described previously 30.

### Detection of S100A4 expression in mouse liver

To detect the expression of S100A4, slices from frozen mouse liver tissues were generated with 10 µm thickness and placed into precooled tubes at -20°C. RNA was isolated after adding lysis buffer and subsequent sonication at 50% for 5 pulses using the DNA/RNA/protein kit (Roboklon, Berlin). The isolated RNA was used to measure the expression levels in a qRT-PCR as described for the expression analysis.

### *In vivo* tolerability for E12

A Maximum Tolerated Dose (MTD) test was carried out in collaboration with the EPO GmbH Berlin-Buch in compliance with the United Kingdom Coordinated Committee on Cancer Research guidelines and was authorized by the Landesamt für Gesundheit und Soziales (LaGeSo), Berlin, Germany (Approval number E0023/23). The study was performed using 6-8 weeks old female SCID beige mice obtained from the Janvier Lab. The mice were randomly assigned to 4 groups (2 animals/group). Concentrations of 25, 50, 100, 200 mg/kg of E12 were applied orally (p.o.) daily to the mice for 5 consecutive days. The mice were observed daily, and the body weight was recorded until study termination. After study termination, animals were evaluated by gross necropsy for potential changes in the major organs.

### *In vivo* testing of E12 in the HCT116 CRC metastasis model

A xenograft transplantation experiment was carried out in collaboration with the EPO GmbH Berlin-Buch in compliance with the United Kingdom Coordinated Committee on Cancer Research guidelines and was authorized by the Landesamt für Gesundheit und Soziales (LaGeSo), Berlin, Germany (Approval number E0023/23). 6-8 weeks old female SCID beige mice (Charles River, Sulzfeld) were used for intrasplenic transplantation of 3 × 10^5^ HCT116/CMVp-Luc cells in anesthetized mice.

The mice were assigned to 2 groups randomly (10 animals/group). For therapy, 25 mg/kg of E12 was applied orally (p.o.) daily. Control animals received solvent. The tumor growth and metastasis formation in the liver was monitored by non-invasive bioluminescence imaging (BLI, using 150 mg/kg D-luciferin in PBS, Biosynth, Staad) in anesthetized mice using the imaging system NightOWL LB 981 (Berthold Technologies, Bad Wildbad). The tumor growth and metastasis formation were imaged and quantified by IndiGo software version 2.0.5.0 (Berthold Technologies, Bad Wildbad). After termination of the experiment, the transplantation site organ, the spleens and the target organ of metastasis, livers, were removed and cryopreserved in liquid nitrogen.

### *In vivo* pharmacokinetic study

6-8-weeks old female SCID beige mice (Charles River, Sulzfeld) were used for studying the pharmacokinetics of E12, which was carried out in collaboration with the EPO GmbH Berlin-Buch in compliance with the United Kingdom Coordinated Committee on Cancer Research guidelines and was authorized by the Landesamt für Gesundheit und Soziales (LaGeSo), Berlin, Germany (Approval number E0023/23). Mice were treated with a single dose of 25 mg/kg of E12 and blood was drawn at specific time points, with 2 animals per timepoint. The timepoints were 15, 30, 60 min, 2, 4, 6, and 24 h. The plasma samples were diluted 1:10 in methanol, centrifuged and the supernatant was used to measure the concentration of E12. A standard curve of E12 ranging from 0.5 to 500 ng/ml was used for quantification. The analysis was performed on a Vanquish Horizon HPLC coupled to a Quantiva mass spectrometer (Thermo Fisher Scientific Inc, Waltham). For injection, 1 uL of sample was used. The mobile phases used were water (buffer A) and acetonitrile (buffer B), both containing 0.1% formic acid. The proportion of buffer B changed from 45% to 100% in 4 minutes, left at 100% for other 2 minutes and reconditioning with initial conditions for other 2 minutes. Two transitions for the compound E12 were used for detection and quantification (m/z 456 → 179 and 456 → 213). The analysis was performed in positive mode. Quantification was performed using the software QuantBrowser (Thermo Fisher Scientific Inc, Waltham). The mass spectrometry was done by Guido Mastrobuoni from the Proteomics and Metabolomics platform in the Berlin Institute for Medical Systems Biology (BIMSB), Max Delbrück Center for Molecular Medicine in the Helmholtz Association, Berlin, Germany.

### Statistical analysis

Statistical analyses were performed with GraphPad Prism version 8.0.2 and 10.4.1 (GraphPad Software Inc, La Jolla, CA). One-way analysis of variance (ANOVA) with Dunnett's post-hoc-test was used for comparison of the control group (DMSO) with multiple compound-treated groups. In addition, Gaussian distribution was confirmed using the Kolmogorov-Smirnov test. For the calculation of the Half Maximal Inhibitory Concentration (IC_50_), a sigmoidal dose-response - Inhibition curve fit was performed, with a baseline defining 0% viable cells determined via using a 10% DMSO positive control. This analysis therefore resulted in the calculation of an absolute IC_50_, which was used due to widely varying maximum toxicities of the selected compounds. For the regression analysis, all compound concentrations were transformed into a logarithmic form and normalized to the control group. The Half Maximal Effective Concentration (EC_50_) was defined by the lowest compound concentration that led to at least 50% inhibition of S100A4 mRNA expression. The IC_50_ and EC_50_ values were then used to calculate the Therapeutic Index (TI) as described in equation 1.

TI = EC_50_/IC_50_ (1)

For the comparison of dose-response effectiveness and toxicity, S100A4 mRNA values from qRT-PCR and viability values of MTT assay were used for calculation as described in equation 2 and 3.

S100A4 inhibition [%] = (1-S100A4 mRNA expression) * 100% (2)

Toxicity [%] = (1-cell viability) * 100% (3)

Dose-response curves for concentration dependent visualization of efficacy, potency and toxicity were created using variable slope (four parameter) analysis for nonlinear regression (curve fit).

## 3. Results

### HTS identifies novel transcriptional S100A4-inhibiting compound candidates

**Figure 1:**
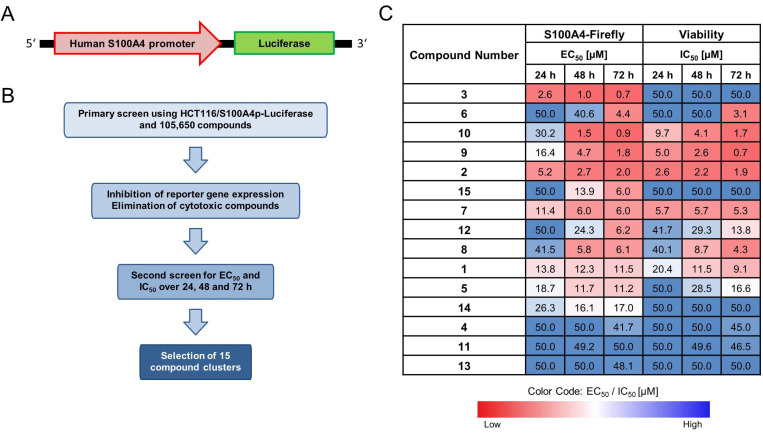
Identification of novel small molecule transcriptional inhibitors for S100A4. (**A**) Scheme of HCT116/S100A4p-luciferase reporter construct. Luciferase gene is driven by the human S100A4 promoter (S100A4p). (**B**) Scheme of the steps for the HTS conducted at the EMBL in Heidelberg. (**C**) Table of the best hits from 15 different chemical clusters. HTS results showing the required concentrations in μM for 50% inhibition of the firefly reporter signal (EC_50_) and 50% reduction in cell viability (IC_50_) for 24 h, 48 h and 72 h. The color code represents the concentration required for each EC_50_ and IC_50_, with red for lower and blue for higher concentrations.

The HTS of 105,600 compounds from the screening library identified potential candidates for transcriptional inhibition of S100A4 (Figure 1). The best hits of 15 different clusters with the strongest average inhibition of the firefly luciferase expression are referred in this work as compounds E1-E15 (Figure 1C, Table S1). The hit clusters resulting from the HTS were arranged according to their chemical structure using established standard workflows of 2D-fingerprints of the CBCF from EMBL. This categorization led to 15 distinct chemical clusters or singletons (13 and 14) with 2-9 chemically similar compounds within each cluster. Moreover, Clusters 3,4,5,6,9,10 and 11 consisted of 2 similar chemical compounds each. Clusters 1,2,7, and 8 had 3 chemically similar members each. Cluster 12, which harbors the most potent compound found in this study, E12, with 6 other structurally related compounds and cluster 15, which consists of 9 chemically similar compounds. The most active hit candidate of each cluster was tested in the first round of evaluation for additional confirmation within this study, and the common scaffold, which was the basis for the clustering, are shown in Table S1. Based on this HTS, E3 shows the lowest EC_50_ for firefly signal inhibition for all treatment time points (0.7 µM - 2.6 µM) and has an IC_50_ for viability inhibition greater than 50 µM. Other compounds (E2, E7, E8, E9, E10, E12, E15) also show low EC_50_ values after 48 h or 72 h of treatment, with different IC_50_ values.

### E2, E10 and E12 are the most promising novel transcriptional S100A4-inhibiting leads

To validate the inhibitory properties of the novel compounds, a comprehensive analysis was performed to assess their capabilities to inhibit S100A4 transcription. All compounds were administrated to HCT116 cells, which have a medium S100A4 expression, for 24 h and 48 h, and to SW620 cells, which have a high S100A4 expression 60, for 24 h at concentrations of 1 µM, 10 µM, and 30 µM, with S100A4 mRNA expression levels quantified via qRT-PCR. The summary of the data for 10 µM (Table S1) indicates that several compounds demonstrated limited efficacy in reducing S100A4 mRNA levels, with E1, E5 and E11 showing reductions of less than 15%, while E4, E8 and E9 led to increased S100A4 mRNA expression after a 24 h or 48 h of treatment. In contrast, E6, E7, E10, E12 and E15, exhibited a pronounced reduction of S100A4 mRNA levels already after 24 h. Other compounds, such as E2, E3, E13 and E14, demonstrated stronger reductions only after 48 h of treatment. In the SW620 cell line, the compounds were generally less effective, with only E12 showing a strong inhibition of S100A4 mRNA expression. Given the fact that the HCT116 cell line was chosen as the primary test system for HTS, the selection of the potential leading candidates focused primarily on their performance in this cell line. The selection criteria of the three most promising compounds for further evaluation were based on two key factors: firstly, the strongest reduction of S100A4 mRNA level at 10 µM after 48 h of treatment, and secondly, a concentration- and time-dependent reduction of S100A4 mRNA expression (Table S2 for 1 µM and Table S3 for 30 µM data). Through this initial re-screening of the 15 identified HTS hits, E2, E10, and E12 emerged as the most effective and potent inhibitors of S100A4 mRNA expression. Consequently, the following experiments focused on the evaluation of these compounds.

### Selected compounds inhibit the S100A4 mRNA and protein expression

**Figure 2:**
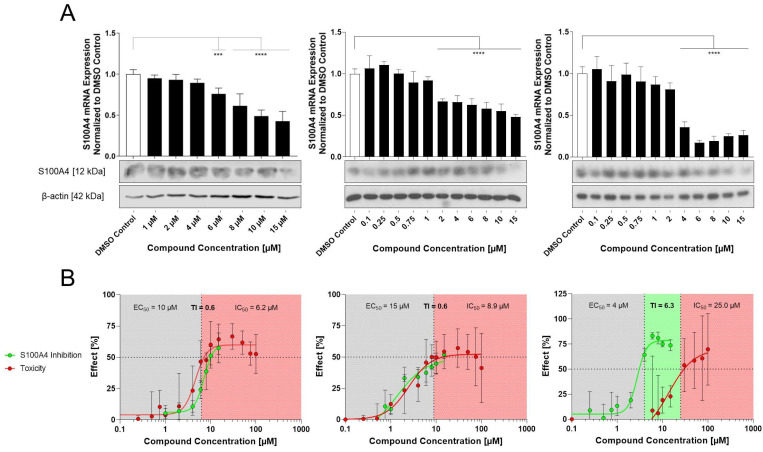
S100A4 inhibition in relation to the toxicity of the selected compounds. (**A**) Effect of E2 (left), E10 (middle) and E12 (right) on the S100A4 expression in HCT116 cells after 24 h of treatment with a broad range of concentrations from 1-15 µM for E2 and 0.1-15 µM for E10 and E12. S100A4 mRNA expression is relativized to DMSO control (DMSO = 1). DMSO (white bar) represents negative control for untreated cells. Each diagram shows means and error bars (95% confidence intervals) calculated from 3 independent experiments. Statistical significance was performed with ordinary one-way ANOVA using GraphPad prism 10.4.1 and multiple comparison was done by Dunnett's post-tests (* = p < 0.05; ** = p < 0.01; *** = p < 0.001; **** = p < 0.0001). S100A4 protein expression was determined by Western blot under the same treatment conditions with ß-actin as a protein loading control. (**B**) Therapeutic margin and TI for compounds E2 (left), E10 (middle) and E12 (right), represented via displaying the compounds' S100A4 inhibition and toxicity with their calculation based on the data generated for HCT116 cells after 24 h of treatment (qPCR and MTT assay). The grey area denotes compound concentrations resulting in S100A4 inhibition below 50%, the green area indicates the therapeutic margin between the EC_50_ and IC_50_, and the red area denotes compound concentrations leading to a reduction in viability of more than 50%. The means and error bars representing 95% confidence intervals from at least two independent experiments.

To achieve a more precise characterization of the concentration-dependent inhibition of S100A4, the selected compounds (E2, E10, E12) were further evaluated over an extended concentration range (1-15 µM for E2 and 0.1-15 μM for E10 and E12) at both the mRNA and protein level following a 24 h treatment (Figure 2A). All 3 compounds reduce the S100A4 mRNA expression in a concentration-dependent manner. E2 showed significant inhibition at concentrations starting from 6 µM (EC_50_ = 10 µM). At the protein level, reduction of S100A4 was only visible at 15 µM, demonstrated by Western blot. E10 and E12 exhibited higher potency and more pronounced inhibitory effects. E10 showed significant reduction in S100A4 mRNA expression at concentrations as low as 2 µM (EC_50_ = 15 µM). However, a reduced protein expression could be seen after 24 h only at the highest concentration of 15 µM in the Western blot. By contrast, E12 reached a maximum of inhibition of 75% at 6 μM, with the lowest EC_50_ of the selected compounds (EC_50_ = 4 µM). S100A4 protein level analysis showed a concentration-dependent inhibition with E12 beginning at 6 µM. These findings confirm the transcriptional inhibitory effect of the selected HTS-identified compounds, as evidenced by the reduction of S100A4 mRNA expression and subsequent protein expression reduction.

### E12 exhibits the highest Therapeutic index (TI) and most favorable impact on cell viability of the selected compounds

To systematically assess the effects of the newly identified compounds on cell viability, an MTT assay was performed. The selected compounds exhibited a concentration-dependent decrease in cell viability (Figure S1). E2 showed a 50% reduction of the cell viability with an IC_50_ value of 6.2 µM after 24 h of treatment, which remained relatively stable at 5.4 µM after 48 h. E10 displayed a similar IC_50_ value after 24 h of 8.9 µM, which decreased to 3.2 µM after 48 h. Further, out of the 3 compounds, E12 exhibited the highest IC_50_ value of 25.0 µM after 24 h, which declined to 7.3 µM after 48 h of treatment in HCT116 cells. By correlating the compound-specific effects on the reduction of viability with their inhibition of S100A4 mRNA expression (Figure 2), the concentration-response-curves enabled a comprehensive evaluation of the therapeutic potential of the novel compounds. This analysis also served as a basis for calculating the TI, a key parameter that represents an easy comparable factor for determining the therapeutic margin of an active compound. For the 24 h treatments in HCT116 cells with the selected compounds, E2 and E10 displayed no discernable therapeutic margin, reflected by their low TI of 0.6. E12 showed the highest TI of 6.3 (Figure 2B). At concentrations of 4 µM and higher, E12 effectively inhibited S100A4 mRNA expression (over 50%) while causing relatively minor decrease in cellular viability. Accordingly, E12 displayed the most promising TI of all 3 selected compounds.

### Compounds E2, E10 and E12 reduce wound healing capacity in HCT116 cells

Next, the selected compounds were applied to a wound healing assay, demonstrating a concentration-dependent inhibition of wound closure. Consistent with its relatively weaker inhibition of S100A4, E2 had the least distinct effects on wound healing, with a concentration of 15 µM inhibiting the wound healing by 39% after 72 h of treatment compared to the DMSO control. In contrast, E10 and E12 showed stronger inhibition of the wound healing capacity of the cells. E10 inhibited wound healing at concentrations as low as 0.5 µM, with a maximum effect observed at 2 µM, resulting in 63% inhibition of the wound healing at 72 h post-treatment. E12 showed a similar inhibition of wound healing at a concentration of 0.5 µM, with wound healing reduced to 27% confluence at 4 µM after 72 h. These findings demonstrate a clear correlation between S100A4 inhibition and the reduction of wound healing capacity, indicating that the selected compounds restrict cell migration and proliferation in a concentration-dependent manner.

### Analogues showed no improved S100A4 mRNA inhibition compared to E12

Compound E12, an N-(4,6-dimethyl-1,3-benzothiazol-2-yl)-2-(4-fluorobenzenesulfonamido)benzamide, was the most effective compound for S100A4 mRNA inhibition identified in an extensive HTS. In addition, it displayed a high TI compared to the other selected compounds which concludes that it will be the most promising candidate for *in vivo* studies. Chemically, the core structure consists of an anthranilic acid, which is decorated with a fluorophenyl-sulfonamide and an amino-benzothiazole group. Interestingly, similar substances containing sulfonyl-benzamides have been reported to exert anti-cancer properties. However, so far, there has been no direct link to S100A4 transcriptional inhibition 31,32. The calculated physical-chemical properties 33 predict a high degree of drug-like properties for E12, adhering to the Lipinski rule of five 34 for orally absorbable small molecules likely to exhibit an acceptable oral bioavailability.

**Figure 4:**
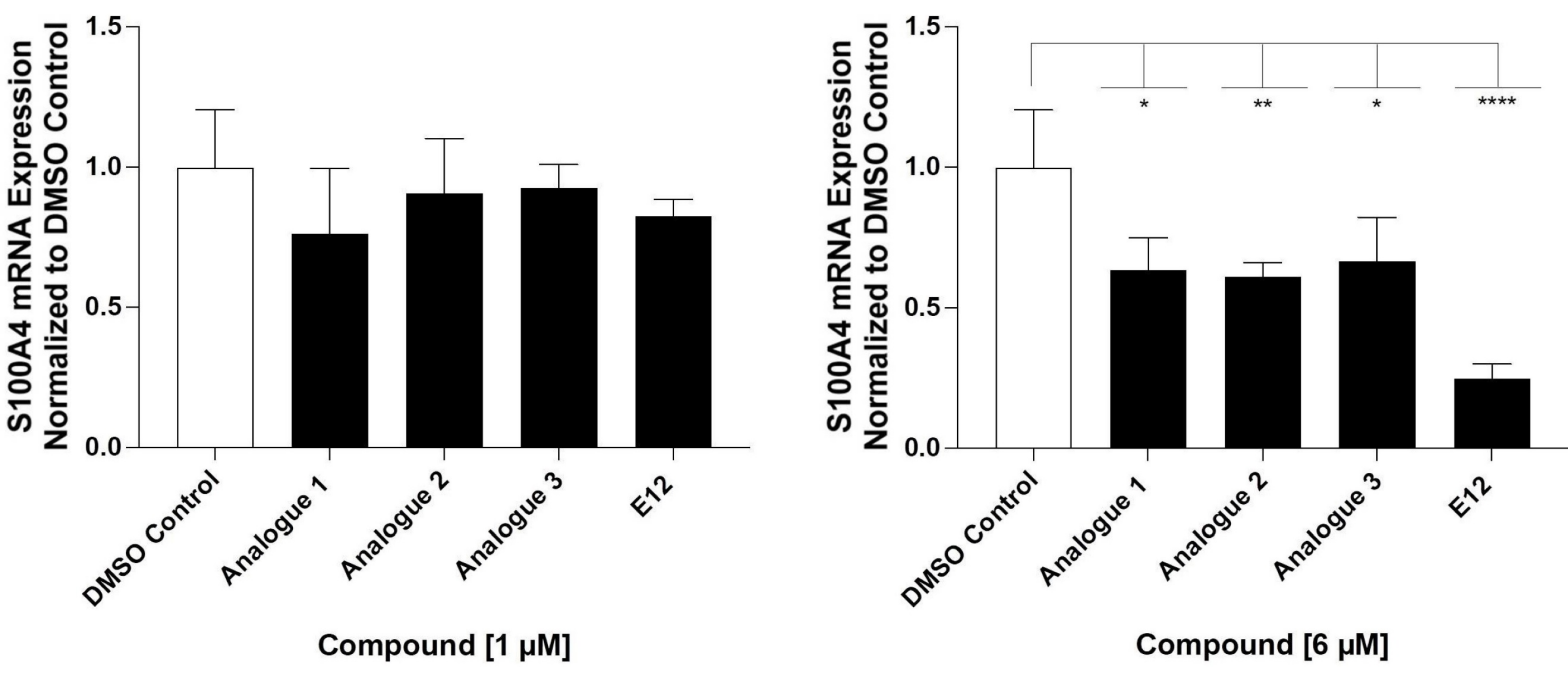
Inhibitory effect of analogues A1, A2 and A3 on S100A4 mRNA inhibition after 24 h of treatment at 1 µM (left) and 6 µM (right) compared to DMSO control and E12 control. DMSO (white bar) represents negative control for untreated cells. E12, which represents the reference control, and the experimental conditions are depicted in black. Each diagram shows means and error bars (95% confidence intervals) calculated from 3 independent experiments. Statistical significance was performed with one-way ANOVA and multiple comparison was done by Dunnett's post-tests using GraphPad prism 10.4.1 (* = p < 0.05; ** = p < 0.01; *** = p < 0.001; **** = p < 0.0001).

Considering that the tested compound E12 was derived from chemical clusters of the HTS, 3 structural analogs of E12 were selected to test for improved performance in either potency or cell viability. All analogues of E12 named Analogue 1 (A1), Analogue 2 (A2) and Analogue 3 (A3) were tested in HCT116 cells at 1 and 6 µM over 24 h to observe their S100A4 mRNA inhibitory capacity. In the case of A1, the fluor in the phenyl group of E12 was exchanged by an ethoxy group and the amino-benzothiazole group only harbours one methyl group. Further, for A2 the fluor was exchanged by an ethoxy group as well and the benzothiazole group is replaced by a thiazol connected to a fluorophenyl group. In A3 the amino-benzothiazole group harbours one methyl group and the sulfonamide is replaced by a carboxyl group connected to the phenyl which is decorated by one methyl group. Each experiment included a treatment of E12 at 1 µM and 6 µM as a reference control. For the 1 µM treatments, A1, A2 and A3 reduced the S100A4 mRNA expression by 24%, 10% and 8% respectively; the corresponding E12 treatment showed to reduce the mRNA expression by 18% (Figure 4) with no statistically significant reduction from any compound at 1 µM. For the 6 µM treatments, A1, A2 and A3 reduced the S100A4 mRNA expression by 37%, 39% and 34% respectively; the corresponding E12 treatment showed a reduction of 74% at 6 µM (Figure 4). The 24 h treatment therefore revealed that none of the analogues showed an improvement in S100A4 mRNA inhibition. The treatment with 6 µM clearly showed that a further reduction of S100A4 mRNA expression by at least 50% was not achieved with any of the analogs compared to the DMSO control. This clearly indicates that E12 has the best efficacy to inhibit S100A4 mRNA expression.

### Analogues showed varying effect on cell viability compared to E12

The cellular viability of the analogues was tested to assess their toxicity compared to E12. All MTT assays were conducted in HCT116 cells over 24 h measuring viability as the IC_50_ value. The IC_50_ of A1 was 87.0 µM, which is 6-fold higher than that of E12 as per Table 2. A2 showed to have an IC_50_ value of 46.1 µM three-fold higher than of E12. Conversely A3 had a corresponding IC_50_ value of 14.5 µM; this IC_50_ is 10.5 µM lower than that of E12, suggesting slightly higher toxicity to cells. After this brief evaluation of the analogues A1-A3 compared to E12, it was shown that even though the analogues show a lower effect on viability, they did not outperform E12 in S100A4 mRNA reduction. Therefore, E12 was evaluated further in the following sections.

### E12 reduces S100A4 mRNA and protein expression under various conditions

**Figure 5:**
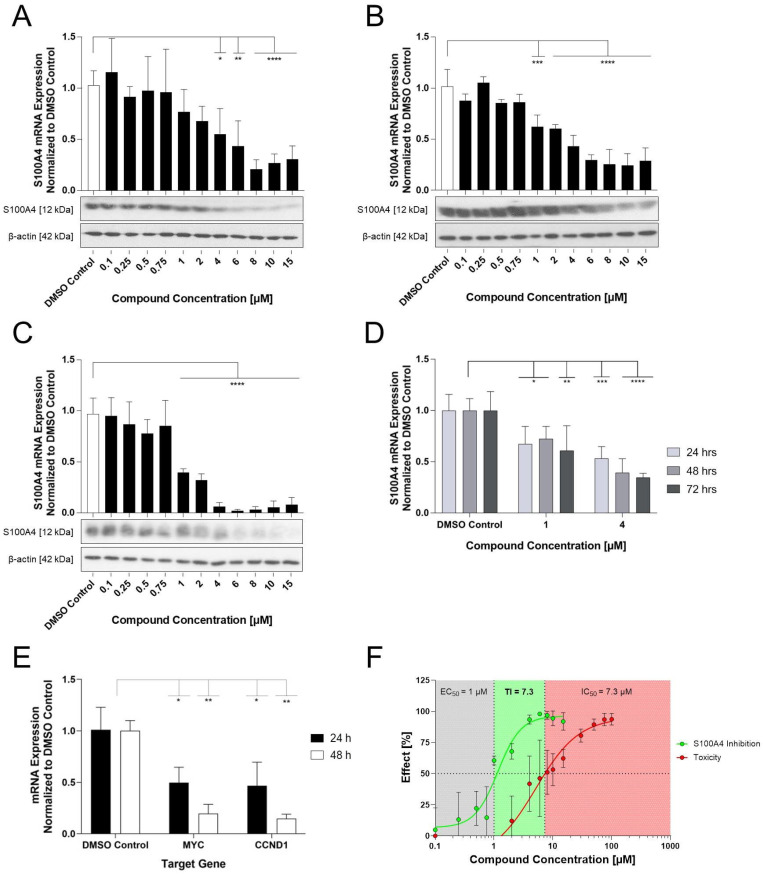
Inhibition of the S100A4 mRNA, protein and Wnt-signaling target gene expression after treatment with E12. S100A4 mRNA and protein expression after (**A**) 24 h and (**B**) 48 h of treatment in SW620 cells and (**C**) 48 h of treatment in HCT116 cells with concentrations ranging from 0.1-15 µM. (**D**) S100A4-mRNA expression after serial time treatments in HCT116 cells at a concentration of 1 µM and 4 µM. (**E**) Effect of E12 at mRNA expression levels of Wnt-signaling associated genes MYC and CCND1 after 24 h and 48 h at 6 µM. (**F**) TI for E12 after 48 h of treatment calculated by the EC_50_ value for S100A4 mRNA inhibition and IC_50_ value calculated by the 48 h MTT data. mRNA expression was normalized to a DMSO control (DMSO = 1). DMSO (white bar) represents negative control for untreated cells. The graph shows means and error bars (95% confidence intervals) calculated from 3 independent experiments. Statistical significance was performed using ordinary one-way ANOVA for (B-D), two-way ANOVA for (A, E) and multiple comparison was done by Dunnett's post-tests using GraphPad prism 10.4.1 (* = p < 0.05; ** = p < 0.01; *** = p < 0.001; **** = p < 0.0001).

The impact of E12 on S100A4 mRNA and protein levels were further evaluated over a broad concentration range (0.1-15 μM) following 24 and 48 h treatment in both HCT116 and SW620 cell lines (Figure 5A-C). E12 reduced S100A4 mRNA expression in a concentration-dependent manner in both cell lines over each period; significant inhibition at the mRNA level after 48 h started from 1 µM treatment onwards with 61% in HCT116 and 38% inhibition in SW620 cells (Figure 5C, B). The strongest inhibition observed after 48 h was at 6 µM in HCT116 (97% inhibition, Figure 5C) and 8 µM after 24 h and 10 µM after 48 h in SW620 cells (73% and 75% inhibition respectively, Figure 5A-B). A similar concentration-dependent effect was translated into the protein expression. Fading protein bands can be witnessed for SW620 cells starting from 6 µM for 24 h and from 8µM for 48 h treatment. For the 48 h treatment duration in HCT116 cells, a reduction of the protein band is visible starting from 6 µM. The next step in the transcriptional inhibition analysis of E12 was its impact on the S100A4 mRNA expression levels following serial treatment in HCT116 cells at 1 µM and 4 µM (Figure 5D). As can be seen in Figure 5D, S100A4 expression was reduced by 32% after 24h treatments at 1 µM; upon continuous application of the compound at this concentration, the expression reduction was maintained at 27% and 39% over 48 and 72 h respectively. 4 µM treatments reduced the expression to around 50% compared to the DMSO control after 24 h, with a stepwise decrease of S100A4 expression after 48 h being reduced by 60%. This reduction was maintained at 72 h at 65% and showed that the continual application of the 1 µM and 4 µM concentration did not increase or decrease the expression and the cells were not able to rescue the S100A4 expression under continual treatment of E12. Furthermore, the TI for E12 after 48 h of treatment in HCT116 cells was calculated using metabolism based MTT 48 h assay data as per Figure S1 and the S100A4 mRNA expression results from Figure 5C. The TI is 7.3 over 48 h and therefore increased compared to the 24 h TI for E12 (Figure 5F).

### E12 reduces Wnt-signaling pathway associated genes

To investigate if E12 was potentially interrupting the Wnt-signaling pathway following treatment, the impact of E12 on the mRNA expression of the two Wnt-signaling target genes MYC and CCND1 was investigated in HCT116 cells over 24 and 48 h treatment at 6 µM (Figure 5E). The compound reduced MYC expression in a time-dependent manner by 50% over 24 h and subsequently 80% over 48 h. Furthermore, CCND1 expression reduction followed a similar pattern at 66% reduction after 24 h and 83% reduction after 48 h.

### E12 reduces the metastatic phenotype of CRC cells *in vitro*

**Figure 6:**
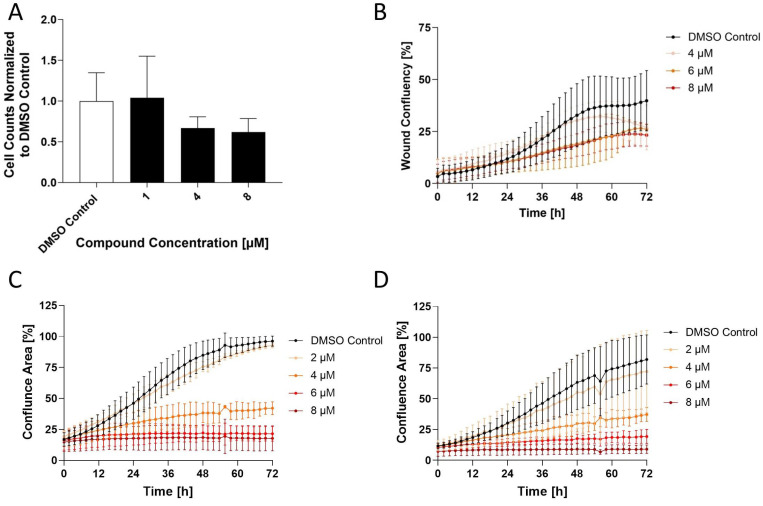
Effect of Compound E12 on migration and proliferation capacity of the HCT116 and SW620 cell lines over 72 h. Wound healing assay observes differences in wound confluency (%) and proliferation is measured in cell confluency (%) both using the IncuCyte Live Cell Imaging system. (**A**) Effect of E12 on migration capacity measured by Boyden chamber assay in HCT116 cells for concentrations 1-8 µM. (**B**) Wound confluence after 72 h of treatment with E12 in SW620 cell line with concentrations ranging from 4-8 µM (**C**) HCT116 cells and (**D**) SW620 cells showed reduction in proliferation following 72 h treatment with E12 ranging from 2-8 µM. Results are shown as mean ± SEM of three independent experiments, performed in triplicates.

The wound healing assay was additionally carried out in the SW620 cell line to assess migration capacity reduction (Figure 6B). A decrease in wound healing capacity to 26.9% of wound confluence was observed after 72 h of treatment, a 32% reduction in relation to the DMSO control (40% wound confluence after 72 h) following treatment with 4 µM. The inhibition of wound healing increased at 6 µM, in particular it can be seen that this reduction was observed earlier after about 24 h of treatment compared to 4 µM, which only started after about 48 h. Treatment with 8 µM resulted in an inhibition plateau with a minimal lower endpoint wound confluence of 23% and therefore no particular greater efficacy.

A second migration assay known as the Boyden Chamber Assay was carried out in HCT116 cells treated at 1, 4 and 8 µM (Figure 6A). At 4 µM, a drop of 33% migration capacity is observed compared to the DMSO control which was further reduced by 8 µM, resulting in a 38% inhibition. Finally, a third phenotypic assay was done in HCT116 and SW620 cells known as the proliferation assay. Using the IncuCyte Live cell imaging system, cell confluency was measured as an indirect correlation for proliferation from time points 0 h to 72 h following a broad range concentration treatment. E12 reduced proliferation in HCT116 cells (Figure 6C) from a concentration of 4 µM onward, reducing cell confluency by 58% and higher concentrations of 6 and 8 µM prevented any proliferation of the cells compared to the DMSO control. The SW620 cell line (Figure 6D) showed a similar pattern of proliferation inhibition following treatment: proliferation was reduced from 4 µM onward, reducing cell confluency by 55% compared to the DMSO control.

### Maximum tolerable dose (MTD) testing of E12 *in vivo*

**Figure 7:**
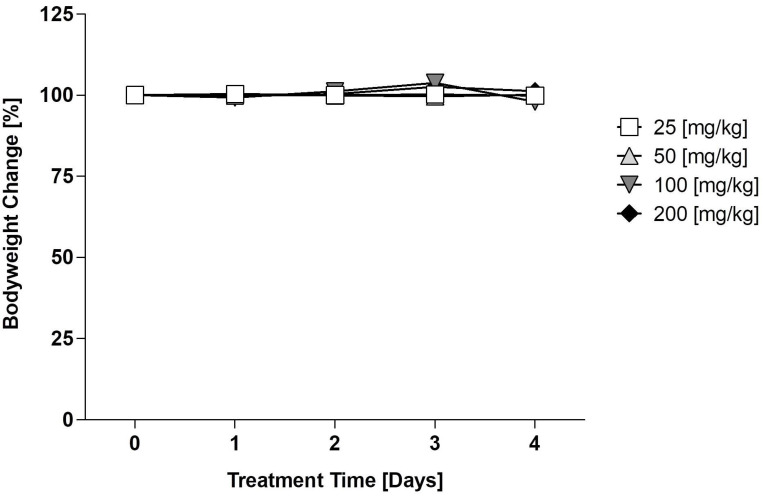
Maximum Tolerable Dose (MTD) evaluation of E12 in mice. The mean change in mice bodyweight following daily treatment over 4 days at ranging concentrations of 25, 50, 100 and 200 mg/kg a day normalized to the bodyweight of the mice on day 0 (100%). The significance threshold was a 10% change in body weight over the 4 days.

The maximum tolerable dose escalation trial carried out by EPO Berlin-Buch was done over 5 days treating four dosage groups (25, 50, 100, 200 mg/kg) every 24 h. The results depicted in Figure 7 demonstrate the bodyweight change in percentage per dosage group over the 5 treatment days normalized to the bodyweight of the mice on day 0. There was no significant reduction or increase in body weight after the 4 days, of which the threshold would be defined as an increase or decrease of body weight by 10%. This indicates that E12 is tolerable for *in vivo* models in an acute manner and can be applied to carry out further *in vivo* testing.

### E12 reduces the metastatic capabilities of HCT116 cells in metastasis model *in vivo*

**Figure 8:**
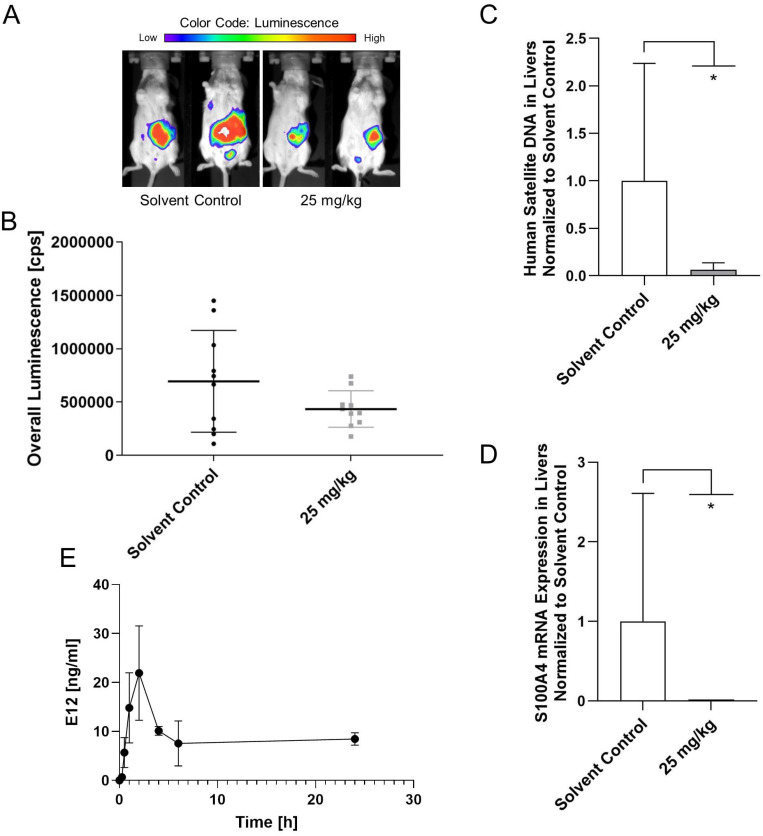
CDX metastasis mouse model for evaluation of the capability of E12 to reduce the metastatic phenotype of HCT116/CMVp-Luc CRC cells. (**A**) *In vivo* imaging of the mice after 21 days of treatment.** (B)** Quantification of the overall luminescence signal of mice. **(C)** Human satellite DNA of the liver of the mice after 25 days of treatment. **(D)** S100A4 expression levels of the liver of the mice after 25 days of treatment. **(E)** Plasma levels of E12 of the 25 mg/kg treated mice over 24 h. The graphs show means and error bars (95% confidence intervals) calculated from 3 independent experiments for the bar graphs and 2 independent samples for the evaluation of the plasma levels. Statistical significance was performed with ordinary one-way ANOVA and multiple comparison was done by Dunnett's post tests using GraphPad prism 8.0.2 (* = p < 0.05; ** = p < 0.01; *** = p < 0.001; **** = p < 0.0001).

After testing for the maximum tolerable dose of E12, a metastasis mouse model was applied using HCT116/CMVp-Luc cells which were intrasplenically transplanted. 20 female SCID/beige mice were assigned to 2 groups: the control group which received solvent and the 25 mg/kg group of E12. Substance and solvent were applied daily p.o. After 21 days of treatment the overall bioluminescence in the mice was quantified (Figure 8A). It can be seen in Figure 8B that E12 reduced the overall bioluminescence of the 25 mg/kg treated mice compared to the solvent control group. The molecular analysis of human satellite DNA and the S100A4 expression in the liver of the mice after 25 days showed a significant reduction in the treatment group for both analyses compared to the solvent control (Figure 8C, D). This suggests that E12 can reduce S100A4 expression and metastasis formation *in vivo*. Further, a brief pharmacokinetic study was conducted measuring the amount of E12 in the blood of 2 SCID/beige mice receiving 25 mg/kg. A spike of 21.9 ng/ml is visible after 2 h in Figure 8E. The amount of E12 drops down to 8.4 ng/ml after 24 h, showing that E12 was successfully absorbed into the plasma of the mice.

## 4. Discussion

This study identified a novel S100A4 transcriptional inhibitor E12, a 2-(4-fluorobenzenesulfonamido)benzamide-based compound that shows high efficiency in reducing the metastatic potential of CRC cells. Thereby, this compound provides a novel efficacious option for targeted therapy for clinical applications of both pre-metastatic and metastatic patients. E12 outperformed all other drug candidates identified in the HTS as well as the chemical analogues tested on the mRNA expression level. This compound demonstrated the highest efficacy of S100A4 expression inhibition at both mRNA and protein levels, and cellular viability assays determined low toxicity of the compound. Subsequent restriction of the metastatic phenotype *in vitro* by E12 was demonstrated; hereby, migration and proliferation capacities of CRC cells were significantly reduced. Further assessment through *in vivo* studies revealed E12 to reduce the metastatic potential of the human CRC cell line HCT116 in a CDX metastasis mouse model. These described characteristics of high efficacy and potency in tandem with low toxicity primes this compound to have a favorable therapeutic margin and holds great promise for application to patients in the future.

One of the key aspects in drug discovery is the identification of a compound that competently executes its function whilst remaining tolerable for the patient. In this regard, E12 proved itself as a functional anti-metastatic compound that is lowly toxic to cells. This is reflected by the TI, a quantitative measurement of the compound's reduction of cell viability exhibited by the IC_50_ value and its efficacy in reducing S100A4 mRNA expression, reflected by the EC_50_ value. The E12 TI score of 6.3 after 24 h treatment represents the broadest therapeutic margin out of the candidates tested. Furthermore, the score of 7.3 after 48 h treatment reflects maintenance of this ratio over time, highlighting the compound's preservation of its potential for therapeutic application. The high TI is attributed to E12 reducing S100A4 mRNA expression by 50% at concentrations as low as 4 µM after 24 h, whilst in parallel only exhibiting an IC_50_ value of 25.0 µM. The TI is contingent upon factors such as body mass, biological sex, and other physiological variables 35, and therefore whilst the *in vitro* data suggests a preliminary TI, it cannot be directly inferred to a patient context. However, any compound falling below 10 is considered a “Narrow TI drug (NTID)” 36, and it can therefore be hypothesized that E12 falls in this category. This category is comprised of drugs with a high risk to reward ratio when the patient's life is at risk, such as with chemotherapeutics 37. Metastatic cancer patients are inevitably viewed to be placed in this critical condition bracket. When E12 is ranked in relation to other currently clinically trialed anti-S100A4 transcription inhibitory drugs such as niclosamide, which possesses an EC_50_ of 1 µM and an IC_50_ of 2.2 µM after 24 h of treatment 38, E12 shows a substantial improvement in safety to efficacy ratio. Nevertheless, this compound will need an intensive structure activity relationship study, since E12 and its 2-(4-fluorobenzenesulfonamido)benzamide-based structure hold promise as a lead to further increase its efficacy while simultaneously reducing its toxicity through further medicinal chemistry studies.

The transcription of S100A4 has high relevancy in promoting the execution of CRC metastasis through intracellular and extracellular mechanisms, as well as to modulate the tumor microenvironment to a pro-metastatic state 19-20. Therefore, the significant reduction of S100A4 gene expression by E12 under various treatment conditions holds high potential for treating patients with a high-risk for metastasis. E12 impact on S100A4 mRNA, protein and metastatic phenotype inhibition was effective in both colon cancer cell lines HCT116 and SW620. HCT116 harbours a different genetic profile to SW620, containing a three-base deletion in exon 3 (Ser45) in CTNNB1 and a correlating WT APC gene, whilst the SW620 cell line carries mutations in the APC gene and has a wild-type CTNNB1 gene 39,40. Both mutations result in an aberrant canonical Wnt-signaling pathway, and hence increased S100A4 expression 41-44. This dual effect indicates the compound's ability to treat different mutations that may lead to S100A4 overexpression. The ability to treat patients with high tumour heterogeneity is pivotal in metastasis therapy due to its prominent role in drug resistance 45. It should be noted that SW620 exhibits a more metastatic phenotype, most likely due the cell line's origin from a lymph node metastasis 46. This result greatly supports the use of this compound to treat metastatic stage IV patients, as SW620 thoroughly reflects a metastatic cell type. Concurrently, E12's effect on the primary tumour derived HCT116 cell line indicates its application potential in patients at risk of cancer dissemination (stages I-III). This is particularly relevant when considering that 50% of all patients diagnosed in stages I-III will undergo metastasis throughout the time course of their disease 47. Thus, it can be presumed that E12 could be used clinically in a preventive setting to treat patients that are pre-metastatic (for prevention) and metastatic (for reduction and restriction). In addition, E12 showed to reduce mechanosensory-driven and chemotaxis-driven migration. These are both important mechanisms to reduce dissemination of cancer cells to the pre-metastatic niche. Notably, E12 reduced migration more efficiently in the wound healing assay than in the Boyden chamber assay. We hypothesize that this may be a result of the compound reducing S100A4-induced migration only later in the experiment, after about 12 h to 18 h of treatment. Therefore, the effect of E12 would occur to a lesser extent compared to the wound healing assay, as the Boyden chamber assay is terminated after 18 h. This hypothesis is based on the wound healing migration inhibition beginning 18 h post treatment and peaking at 60 h post treatment at 4 µM and 8µM as well. Any wound healing inhibition prior to 18 h is hypothesized to be due to proliferation reduction which started almost immediately at these concentrations as shown in the proliferation assay. It should be noted here that S100A4 is not known to directly play a role in proliferation, and we hypothesize proliferation is reduced by E12 here through general Wnt-signaling disruption. This would then result in the demonstrated cyclin d1 (CCND1) and C-Myc (MYC) gene expression reduction, two target genes of the Wnt-signaling pathway 48,49 that are known to drive proliferation. For context, Cyclin D1 is an allosteric master regulator of the cell cycle, more specifically it works in concert with the activating cyclin-dependent kinases (CDK4/6) to allow for G1/S checkpoint progression 50,51 and hence tumour growth. The key oncogenic transcription factor C-Myc drives cancer by promoting proliferation and survival 52-54. Reduction of expression of these two oncogenes is not only indicative of Wnt-signaling pathway interruption, but also reflective of the anti-cancerous properties of E12. Notably, this data indicates the possibility that E12 is applicable in treating not only CRC, but also in a wider range of clinical contexts such as other S100A4-prognostic cancers that display aberrant Wnt-signaling pathways, such as breast, ovarian, lung, gastric and melanoma cancer 55-59. Furthermore, this reduction of proliferation by an anti-metastatic compound plays a crucial role in inhibiting the establishment of metastasis in peripheral organs.

The *in vivo* CDX metastasis mouse model experiment has shown that lower concentrations of E12 causes no toxic side effects in mice after 25 days of treatment, while influencing the metastasis formation as seen in the group that received 25 mg/kg of E12. This reduction of overall luminescence after 21 days compared to the solvent treated control, as well as the reduction in human satellite DNA in the liver observed in the treated group compared to the solvent control group after 25 days, can conclude E12's efficacy *in vivo* in reducing the formation of liver metastases. The S100A4 mRNA levels in the liver were substantially lower compared to the solvent treated group, demonstrating that the reduction of metastasis is due to the inhibition of the S100A4 gene expression. Further optimization of E12 may increase its efficacy and reduce toxicity to enhance its potential. Moreover, further evaluation of its mode of action and application in different *in vivo* models for optimal dose formulation and application route is the next crucial step.

As the lead candidate identified in this HTS study, the 2-(4-fluorobenzenesulfonamido)benzamide -based compound E12 holds great promise for reducing and restricting the most lethal attribute of cancer: metastasis, and thus can be considered a potential lead hit for optimization and development of a new therapy approach amidst the ongoing anti-metastasis therapy renaissance.

## Supplementary Material

Supplementary figures and tables.

## Figures and Tables

**Figure 3:**
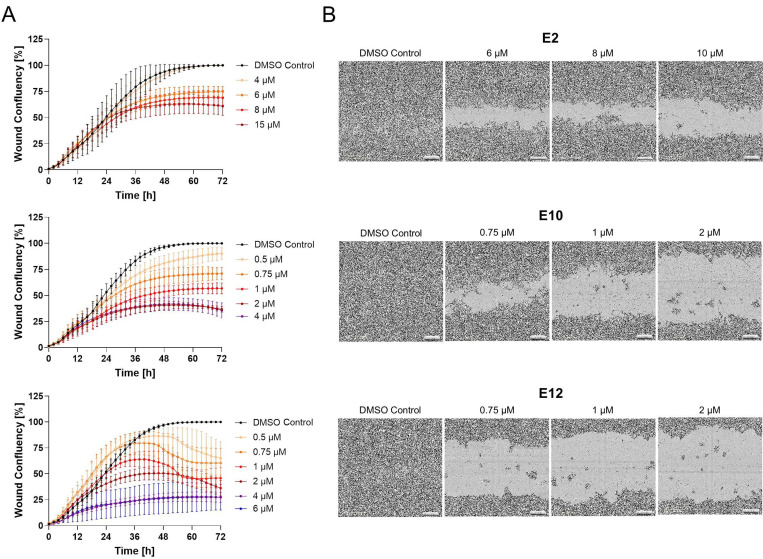
Effect of the compounds E2, E10 and E12 on HCT116 cell wound healing capacity. (**A**) Wound confluence in % monitored over 72 h using the IncuCyte Live cell imaging system. All compounds were tested from 0.1 - 15 µM. E2 displayed with concentrations from 4 - 15 µM (top), E10 with concentrations from 0.5 - 4 µM (middle) and E12 with concentrations from 0.5 - 6 µM (bottom). Mean values were calculated from 3 independent experiments. (**B**) Microphotographs of the wound confluence of HCT116 cells 72 h after treatment with concentrations ranging from 6 - 10 µM for E2 (top) and 0.75 - 2 µM for E10 (middle) and E12 (bottom). Results are shown as mean ± SEM of three independent experiments, performed in triplicates.

**Table 1:** Primer sequences (5'→3') for selected genes and their expression analysis via qRT-PCR

Gene		Primer Sequence
*S100A4*	Forward Reverse	5'- CTC AGC GCT TCT TCT TTC -3' 5'- GGG TCA GCA GCT CCT TTA -3'
*GAPDH*	Forward Reverse	5'- GAA GAT GGT GAT GGG ATT TC-3' 5'-GAA GGT GAA GGT CGG AGT-3'
*MYC*	Forward Reverse	5' CAC CAG CAG CGA CTC TGA-3'
		5'-GAT CCA GAC TCT GAC CTT TTG C-3'
*CCND1*	Forward Reverse	5'-GAA GAT CGT CGC CAC CTG-3'
		5'-GAC CTC CTC CTC GCA CTT CT-3'

**Table 2:** S100A4 mRNA expression levels and cell viability after 24 h analogue treatment of HCT116 cells. S100A4 mRNA expression normalized to DMSO is represented in [%] after treatment with 1 or 6 µM of each compound. Cell viability is shown as IC_50_ in µM and was determined after 24 h of compound treatment displayed in Figure S2.

Compound	S100A4 mRNA Expression Normalized to DMSO control [%]		IC_50_[µM]
	1 µM	6 µM	
Analogue 1 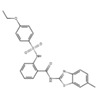	76	64	87.0
Analogue 2 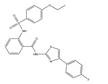	91	61	46.1
Analogue 3 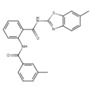	93	67	14.5
E12 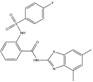	83	26	25

## Abbreviations

ANOVA: analysis of variance
BLI: bioluminescence imaging
CBCF: chemical biology core facility
CDK4/6: cyclin dependent kinase 4/6
CMV: cytomegalovirus
CRC: colorectal cancer
DAPI: 4',6-diamidin-2-phenylindole
DMSO: dimethylsulfoxide
FBS: fetal bovine serum
HTS: high-throughput screen
LaGeSo: landesamt für gesundheit und soziales
Luc: luciferase
MTD: maximum tolerable dose
MTT: 3-(4,5-dimethylthiazol-2-yl)-2,5-diphenyltetrazolium bromide
NTID: narrow TI drug
p.o.: per os
qRT-PCR: quantitative reverse transcription - polymerase chain
TI: therapeutic index
TCF4: transcription factor 4
YSR: year-survival rate
