# Supplementary material for: Novel High-Throughput Screen Identified S100A4 Inhibitors for Anti-Metastatic Therapy
Source: Int J Biol Sci. 2025 Jul 11;21(10):4683–700. doi: 10.7150/ijbs.113805 (PMC12320493; doi:10.7150/ijbs.113805)
Supplement: Supplementary file 1 — Supplementary figures and tables. [file ijbsv21p4683s1.pdf]

Supplements for the Manuscript:

# Novel High-Throughput Screen Identified S100A4 Inhibitors for anti-Metastatic Therapy

**Table S1:** S100A4 expression levels after treatment with the HTS selected compounds after first screening with low concentration range (1 µM, 10 µM, 30 µM). S100A4 mRNA expression normalized to the DMSO control is represented in [%] after treatment with 10 µM of each compound for 24 h and 48 h in HCT116 cells and 48 h in SW620 cells. Selected compounds E2, E10 E12 are marked in light grey with highlighting their strongest reduction of S100A4 mRNA levels for the respective concentration in dark grey.

| **Compound** | **S100A4 mRNA Expression Normalized to DMSO control [%]** | | |
|---|---|---|---|
| | **HCT116 24 h** | **HCT116 48 h** | **SW620 24 h** |
| **E1** 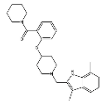 | 89 | 116 | 107 |
| **E2** 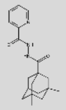 | 85 | **37** | 167 |
| **E3** 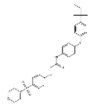 | 105 | 56 | 115 |
| **E4** 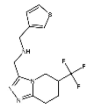 | 122 | 136 | 75 |
| **E5** 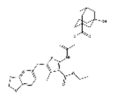 | 102 | 90 | 82 |
| **E6** 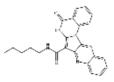 | 78 | 50 | 126 |
| **E7** 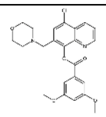 | 81 | 94 | 65 |
| **E8** 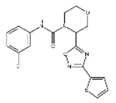 | 118 | 202 | 214 |
| **E9** 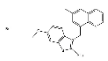 | 208 | 95 | 99 |
| **E10** 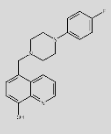 | 49 | **32** | 91 |

| | | | |
|---|---|---|---|
| **E11** 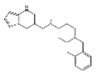 | 98 | 103 | 98 |
| **E12** 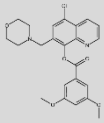 | 4 | **18** | 41 |
| **E13** 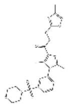 | 97 | 48 | 109 |
| **E14** 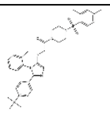 | 89 | 73 | 100 |
| **E15** 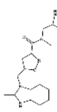 | 81 | 49 | 75 |

**Table S2 and S3:** S100A4 expression levels after treatment with the HTS selected compounds after first screening with low concentration range (Table S2: 1 µM, Table S3: 30 µM). S100A4 mRNA expression normalized to the DMSO control is represented in [%] after treatment with 1 or 30 µM of each compound for 24 h and 48 h in HCT116 cells and 48 h in SW620 cells. Selected compounds E2, E10 and E12 are marked in light grey with highlighting their strongest reduction of S100A4 mRNA levels for the respective treatment concentration in dark grey.

**Table S2:** 1 µM treatment with HTS identified compounds.

| Compound | S100A4 mRNA Expression Normalized to DMSO control [%] | | |
|---|---|---|---|
| | **HCT116 24 h** | **HCT116 48 h** | **SW620 24 h** |
| E1 | 80 | 92 | 83 |
| E2 | 86 | **91** | 94 |
| E3 | 116 | 106 | 110 |
| E4 | 89 | 110 | 77 |
| E5 | 92 | 98 | 85 |
| E6 | 89 | 105 | 81 |
| E7 | 91 | 93 | 88 |
| E8 | 104 | 110 | 117 |
| E9 | 271 | 129 | 90 |
| E10 | 92 | **61** | 77 |
| E11 | 91 | 82 | 98 |
| E12 | 103 | **81** | 102 |
| E13 | 86 | 75 | 101 |
| E14 | 95 | 89 | 97 |
| E15 | 86 | 53 | 92 |

**Table S3:** 30 µM treatment with HTS identified compounds.

| Compound | S100A4 mRNA Expression Normalized to DMSO control [%] | | |
|---|---|---|---|
| | **HCT116 24 h** | **HCT116 48 h** | **SW620 24 h** |
| E1 | 83 | 95 | 60 |
| E2 | 55 | **22** | 113 |
| E3 | 67 | 68 | 107 |
| E4 | 93 | 87 | 80 |
| E5 | 81 | 50 | 48 |
| E6 | 63 | 43 | 117 |
| E7 | 47 | 9 | 104 |
| E8 | 208 | 128 | 207 |
| E9 | 149 | 34 | 67 |
| E10 | 40 | **17** | 99 |
| E11 | 93 | 110 | 104 |
| E12 | 2 | **0** | 34 |
| E13 | 104 | 46 | 95 |
| E14 | 65 | 67 | 88 |
| E15 | 67 | 43 | 121 |

**Table S4:** Overview of HTS identified clusters. The S100A4 expression in the reporter construct of the HTS is shown in % and each compound was tested with 10 µM for 72 h. For compounds not included in the HTS but found through the clustering process an ND for not determined is shown.

| Cluster | Compound Name | Supplier | Supplier ID | MW [Da] | S100A4 expression inhibition in HTS [%] |
|---|---|---|---|---|---|
| E1 | E1 | ChemBridge | 21374804 | 445.596 | 95.1 |
| | E1.2 | | 26693136 | 393.522 | 94.08 |
| | E1.3 | | 38184601 | 412.952 | 75.81 |
| E2 | E2 | Enamine | Z27728028 | 327.421 | 97.92 |
| | E2.2 | | Z27728746 | 299.368 | 96.46 |
| | E2.3 | | Z286454988 | 313.394 | 86.39 |
| E3 | E3 | Enamine | T0503-7182 | 538.655 | 81.65 |
| | E3.2 | | T5337738 | 444.544 | 72.21 |
| E4 | E4 | Enamine | Z1268662935 | 316.345 | 88.65 |
| | E4.2 | | Z1268662690 | 324.344 | 86.37 |
| E5 | E5 | Enamine | T5243227 | 555.639 | 70.81 |
| | E5.2 | | T5271961 | 430.492 | 70.17 |
| E6 | E6 | Enamine | Z1033301156 | 399.445 | 82.26 |
| | E6.2 | | Z1089877546 | 385.419 | 74.18 |
| E7 | E7 | Enamine | Z56836338 | 442.892 | 97.94 |
| | E7.2 | | Z56174876 | 400.831 | 94 |
| | E7.3 | | Z56836325 | 417.285 | 96.96 |
| E8 | E8 | Enamine | Z2034819477 | 390.844 | 88.56 |
| | E8.2 | | Z2034819411 | 381.408 | 82.12 |
| | E8.3 | | Z2234920098 | 408.834 | 64.17 |
| E9 | E9 | Enamine | Z56777163 | 336.431 | 88.56 |
| | E9.2 | | Z56777161 | 350.457 | 81.89 |
| E10 | E10 | Enamine | Z44113219 | 337.391 | 96.01 |
| | E10.2 | | Z44283769 | 333.427 | 91.15 |
| E11 | E11 | Enamine | Z1147801836 | 353.504 | 98.93 |
| | E11.2 | | Z1101327871 | 339.478 | 98.14 |
| E12 | E12 | Enamine | T0505-9066 | 455.525 | 82.4 |
| | E12.2 (A1) | | T0506-7625 | 467.561 | 35.2 |
| | E12.3 | | T0505-7380 | 522.017 | 14.9 |
| | E12.4 | | T0507-7068 | 512.515 | -3.63 |
| | E12.5 (A2) | | T0510-2106 | 497.562 | 69.91 |
| | E12.6 (A3) | | T5401236 | 401.481 | 2.34 |
| E13 | E13 | Enamine | T5320044 | 491.647 | 52 |
| E14 | E14 | Enamine | T5454969 | 617.824 | 27 |
| E15 | E15 | Chembridge | 45775314 | 381.451 | 51 |
| | E15.2 | | 56093530 | 379.412 | ND |
| | E15.3 | | 12793122 | 326.35 | 19.26 |
| | E15.4 | | 92172055 | 370.469 | -21.3 |
| | E15.5 | | 90937896 | 379.436 | ND |
| | E15.6 | | 89711317 | 336.388 | 28.2 |
| | E15.7 | | 64458800 | 361.397 | 23.85 |
| | E15.8 | | 12264395 | 364.401 | 6.31 |
| | E15.9 | | 77568677 | 338.285 | 29.37 |

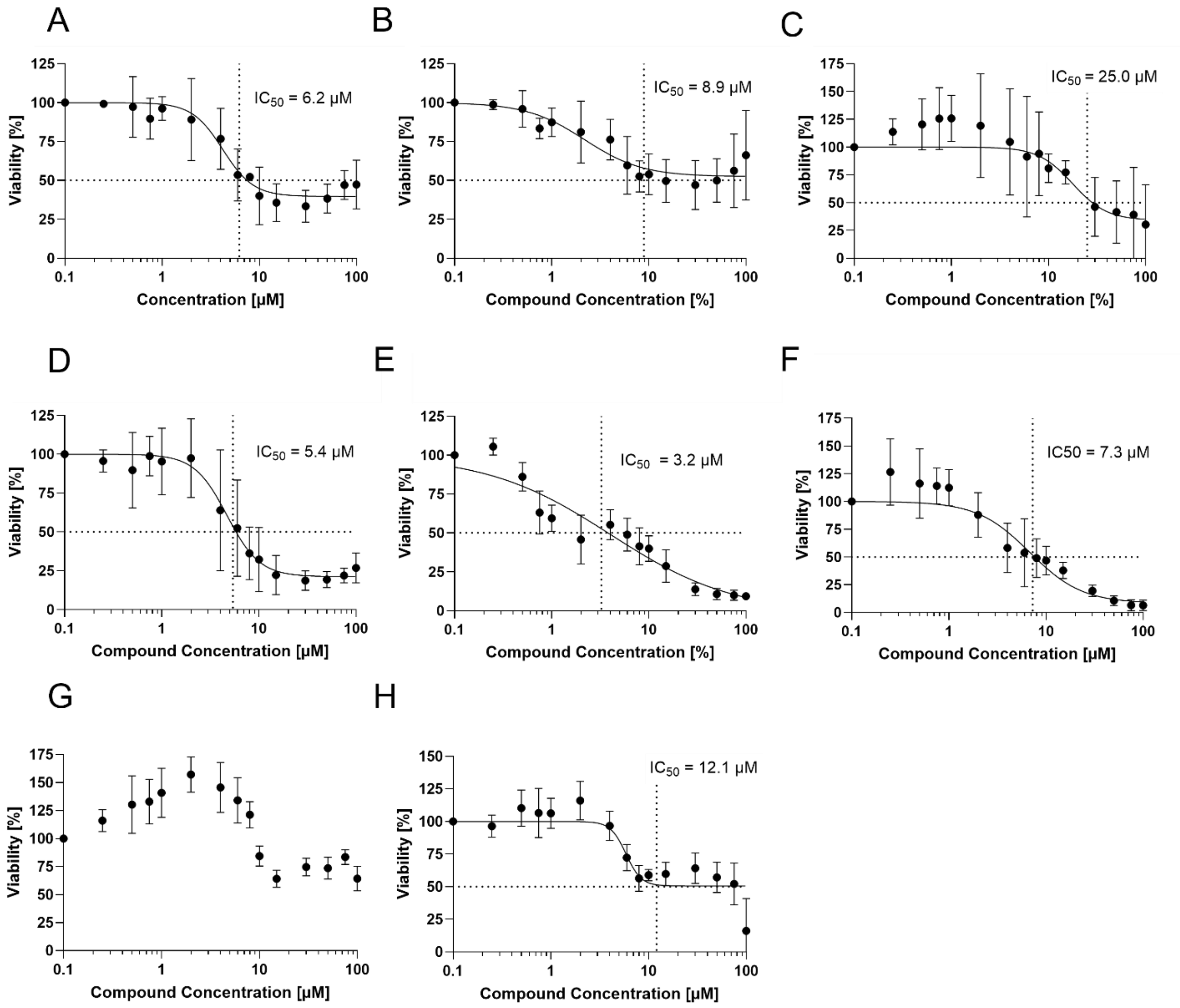


**Figure S1.** To evaluate the effect of the compounds on the cell's viability, a 3-(4,5-dimethylthiazol-2-yl)-2,5-diphenyltetrazolium bromide (MTT) assay was conducted, investigating cellular metabolic activity under compound treatment of E2, E10 and E12 for 24 h (**A-C**) and 48 h (**D-F**) in HCT116 cells and for E12 in SW620 for 24 h (**G**) and 48 h (**H**). For the calculation of the Half Maximal Inhibitory Concentration ($IC_{50}$), a sigmoidal dose-response – Inhibition curve fit was performed, with a baseline defining 0% viable cells determined via using a 10% DMSO positive control. Analysis was conducted with GraphPad Prism version 10.4.1.

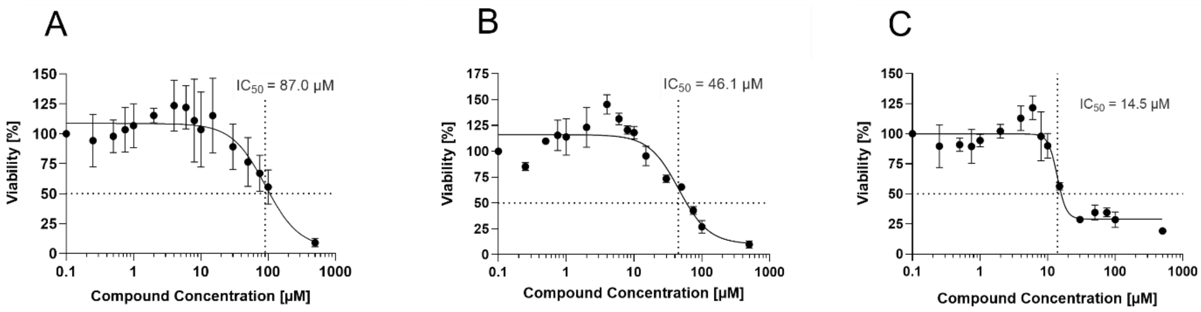


**Figure S2.** To evaluate the effect of the compounds on the cell's viability, a 3-(4,5-dimethylthiazol-2-yl)-2,5-diphenyltetrazolium bromide (MTT) assay was conducted, investigating cellular metabolic activity under compound treatment of A1, A2 and A3 (**A-C**) for 24 h in HCT116 cells. For the calculation of the Half Maximal Inhibitory Concentration ($IC_{50}$), a sigmoidal dose-response – Inhibition curve fit was performed, with a baseline defining 0% viable cells determined via using a 10% DMSO positive control. Analysis was conducted with GraphPad Prism version 10.4.1.
